# Abandonment of nicotine dependence treatment: A cohort study: Abandono do tratamento da dependência à nicotina: Um estudo de coorte

**DOI:** 10.1590/1516-3180.2015.00830309

**Published:** 2016-01-19

**Authors:** Maritza Muzzi Cardozo Pawlina, Regina Cássia Rondina, Mariano Martinez Espinosa, Clóvis Botelho

**Affiliations:** I PhD. Health Sciences, Universidade Federal de Mato Grosso (UFMT), Cuiabá, Mato Grosso, Brazil.; II PhD. Professor of Educational Psychology and Human Development, Department of Educational Psychology, Universidade Estadual Paulista (UNIP), Marília, São Paulo, Brazil.; III PhD. Professor of Statistics, Department of Statistics and Biostatistics, Institute of Public Health, Universidade Federal de Mato Grosso (UFMT). Cuiabá, Mato Grosso, Brazil.; IV MD, PhD. Professor of Pulmonology, Department of Medicine, Universidade Federal de Mato Grosso (UFMT). Cuiabá, Mato Grosso, Brazil.

**Keywords:** Smoking, Treatment refusal, Tobacco use disorder, Tobacco use cessation, Motivation

## Abstract

**CONTEXT AND OBJECTIVE:**

Non-adherence to treatment is one of the hindering factors in the process of smoking cessation. This study aimed to compare sociodemographic characteristics, smoking status and motivation among smokers who maintained or abandoned treatment to stop smoking, and to analyze associations between sociodemographic factors and smoking.

**DESIGN AND SETTING:**

Cohort study on 216 smokers who were attended at healthcare units in Cuiabá, Mato Grosso.

**METHODS:**

The instruments used were the Fagerström, URICA and CAGE questionnaires. Data from the initial evaluation was analyzed using the two-proportion test (α < 0.05). The patients were monitored for six months and those who abandoned treatment were accounted for. Bivariate analysis was conducted, using crude prevalence ratios and 5% significance level (P < 0.05), with abandonment of treatment as the outcome variable. Associations with P < 0.20 were selected for multiple robust Poisson regression (RPa).

**RESULTS:**

The abandonment rate was 34.26%. Males and individuals in the 20-39 age group, in employment, with low motivation, with shorter time smoking and lower tobacco intake predominated in the dropout group. In the final model, gender (RPa 1.47; 95% CI: 1.03-2.10) and age group (RPa 3.77; 95% CI: 1.47-9.67) remained associated with abandonment.

**CONCLUSION:**

Males and individuals in the 20-39 age group, in employment, with low motivation, with shorter time smoking and lower tobacco intake more frequently abandoned the treatment. Male gender and younger age group were associated with abandonment of nicotine dependence treatment.

## RESUMO

**CONTEXTO E OBJETIVOS:**

A não adesão ao tratamento é um dos fatores dificultadores do processo de cessação do tabagismo. Este estudo objetiva comparar características sociodemográficas, *status* do tabagismo e motivação entre fumantes que abandonaram ou não o tratamento do tabagismo e analisar a associação entre fatores sociodemográficos e uso do tabaco.

**TIPO DE ESTUDO E LOCAL:**

Estudo de coorte, com 216 fumantes atendidos em unidades de saúde de Cuiabá, MT.

**MÉTODOS:**

Instrumentos: foram utilizados os questionários Fagerström, URICA e CAGE. Dados da avaliação inicial foram analisados via teste de duas proporções (α < 0.05) e os pacientes foram acompanhados por seis meses, contabilizando-se os que abandonaram o tratamento. Foi realizada análise bivariada, utilizando a razão de prevalência bruta, com nível de significância 5% (P < 0,05); tendo como desfecho o abandono do tratamento. As associações com P < 0,20 foram selecionadas para a regressão de Poisson múltipla robusta (RPa).

**RESULTADOS:**

A taxa de abandono foi de 34,26%. O predomínio no grupo abandono foi do gênero masculino, na faixa etária 20-39 anos, trabalhadores, com baixa motivação, menor tempo de fumo e de carga tabágica. No modelo final, gênero (RPa 1,47; IC 95% 1,03-2,10) e faixa etária (RPa 3,77; IC 95% 1,47-9,67) permaneceram associados ao abandono.

**CONCLUSÃO:**

Indivíduos do gênero masculino, na faixa etária de 20-39, que trabalham, com baixa motivação, menor tempo de fumo e de carga tabágica abandonaram mais o tratamento. Gênero masculino e faixa etária jovem estão associados ao abandono do tratamento da dependência à nicotina.

## INTRODUCTION

Both as a risk factor and as a chronic disease, smoking is one of the great evils of humanity. It has become a worldwide public health problem, since it causes roughly six million deaths a year through direct consumption of tobacco and its derivatives or through exposure to environmental tobacco smoke.^1^

In Brazil, the prevalence of smoking has been declining over the years, ever since public policies were implemented through the National Tobacco Control Program (Programa Nacional de Controle do Tabagismo, PNCT) in 1989.^2^ Currently, this program is called the National Program for Controlling Tobacco and Other Risk Factors for Cancer (Programa Nacional de Controle do Tabagismo e Outros Fatores de Risco de Câncer, PNCTOFR). PNCTOFR is recognized worldwide as one of the most effective programs for controlling tobacco, through its development of multiple actions, and it is an international reference.^3^

The program involves two main action groups: the first one targets prevention of starting to smoke, focusing on children and adolescents; and the second one aims at encouraging smokers to quit smoking.^4^ Tobacco treatment was added to the Brazilian National Health System (Sistema Único de Saúde, SUS) through an agreement with the Tripartite Interagency Commission (Comissão Intergestores Tripartite, CIT), which has created ordinances approving an implementation plan for addressing and treating smoking within SUS, with clinical protocols and therapeutic guidelines for nicotine dependence.^2^

The recommended treatment for smoking is based on psychological support (cognitive-behavioral therapy, CBT) and on the use of medications to control abstinence syndrome.^4^ The consensuses on the treatment of smoking have recommended the medications used by the PNCT as first rate: transdermal nicotine patches, nicotine chewing gum and bupropion hydrochloride.^5^

However, these consensuses have not achieved the desired therapeutic success, with low success rates in clinical trials.^6^ Another problem is the high rates of abandonment during treatment. A high number of patients who are enrolled in programs abandon treatment without having participated in the necessary number of CBT meetings and discontinue their use of medication. These patients are therefore accounted for in surveys as patients presenting treatment failure or relapses during treatment.^7^

The issues surrounding abandonment of treatment have been poorly studied and need to be taken more seriously, given that each patient who gives up the program and continues to smoke will suffer the direct harm caused by tobacco and the impact of morbidity and mortality resulting from diseases related to tobacco consumption. Moreover, abandonment of treatment generates an economic and social burden. Smoking causes an annual loss of R$ 338.6 million to SUS.^8^

In the city of Cuiabá, Mato Grosso, the program functions in accordance with the guidelines and standards of the PNCT, with actions aimed at education and promoting health.^3^ However, one of the difficulties that healthcare professionals find in practice when implementing these actions is that even though the demand for this service is high, the rate of abandonment of treatment during the process of smoking cessation is significant. Some of the hypotheses that can be cited are the level of motivation and the smoker’s occupation and sociodemographic characteristics, which show that there is a need to deepen this understanding in order to propose actions and approaches for treating dependence that are more efficient.

## OBJECTIVE

The objective of this study was to investigate the associations between abandonment of treatment for nicotine dependence and a set of clinical and sociodemographic variables, among a sample of patients seen at healthcare units in the city of Cuiabá, Mato Grosso.

## METHODS

A cohort study was conducted among patients who were over 18 years old and sought or were referred to the smoking cessation programs of four healthcare units in Cuiabá, Mato Grosso (Júlio Müller University Hospital, Campo Velho Healthcare Center and Coxipó and Planalto Polyclinics). All the smokers who enrolled in the initial phase of these programs, from May to August 2012, were invited to participate in this study. Those who agreed were included and their research records were numbered sequentially, thus making up the population of this present study, totaling 216 participants.

The criteria for inclusion were that the subjects needed to be smokers, be over 18 years old and have the desire to quit smoking. These subjects were enrolled in the initial phase of the cessation program. Participants who had cognitive limitations, were dependent on other psychoactive substances, except caffeine, or were pregnant or breastfeeding women were excluded from this study.

In this study, the size of the population (N) and the proportion of smokers who would be able to abandon cigarettes in the city of Cuiabá during the data collection were unknown. Therefore, in order to determine the approximate size of the sample (n), an expression taking the coefficient of reliability to be 95% and the sampling error to be 7.00% (d = 0.07) was used.^9^^,^^10^^,^^11^ This indicated that the distance between the sample estimate and the population parameter should not exceed this value, with a proportion of 0.5 (P = 0.5). This value was used because of what was not known about the prevalence of the outcome, and also because this value provided greater variance and made it possible to obtain a sample of larger size with a given fixed precision.^12^

From using the expression (1), the size of the sample obtained was 196 participants. Considering a percentage loss of 10%, the final sample size was 216 individuals. Thus, all the patients who had enrolled in the programs since May 2012 participated in this study, and the data collection ended when the number of patients needed for the sample was reached.

$$n=\frac{{\left(Z_{\alpha /2}\right)}^{2}p\left(1-p\right)}{d^{2}}$$ (1)

The same treatment protocol was used in this study for all the patients: nicotine replacement therapy (NRT) + bupropion + cognitive behavioral therapy (CBT). The participants who remained in the program were followed up by a doctor during the initial phase, for 30 days after starting to take medication, and at monthly evaluations until completing six months of treatment. After the initial evaluation conducted by a psychologist and after the evaluation instruments had been applied, all of the participants were invited to attend CBT, consisting of four group sessions (each with 10 to 15 patients), lasting one hour and a half, once a week over a four-week period.^4^ Subsequently, there were five follow-up meetings: after 15 days, 30 days, 60 days, 90 days and 180 days.

The instruments used in the initial individual interview with a psychologist for data collection were the following:

1. Sociodemographic profile questionnaire: This was specifically designed for this study and was constructed based on the model used and distributed by INCA/MS (National Cancer Institute, Ministry of Health). It contains two parts: Part I - identification and sociodemographic data, with the following variables: gender, age, marital status, occupation, education and family income; and Part II - status of tobacco use, with the following variables: time spent smoking, number of cigarettes per day, age when smoking started and the number of attempts to quit.
2. Fagerström Test for Nicotine Dependence (FTND):^13^ This is used for analysis on nicotine dependence, such that scores higher than the median (≥ 6) are categorized as having high dependence, and those with values below 6, as having low dependence.
3. CAGE (Cut-down, Annoyed, Guilty and Eye-opener) questionnaire: This was designed for detecting suspected alcoholism. It was developed in 1974^14^ and validated in Brazil in 1983.^15^
4. URICA (University of Rhode Island Change Assessment),^16^ reduced version: This evaluates the motivational stage (precontemplation, contemplation, preparation and action) in relation to drug-using behaviors. The Transtheoretical Model of Behavioral Change (TMBC), based on the internships of the American James O. Prochaska, was validated and standardized for the Brazilian population in relation to illicit drugs, with transcription to tobacco.^17^ A previous study was used as a reference to dichotomize the data into precontemplation/contemplation and preparation/action.^18^

After the instruments had been fully administered, the data obtained were checked and entered twice into the Epidata software, version 3.1. In the present study, the results from the instruments in the initial evaluation were analyzed, and the patients were monitored for six months, taking into account the number of people who abandoned the program. Abandonment was defined as a situation in which after a smoker had attended the medical consultation and the initial evaluation with the psychologist, he or she did not attend the first CBT session or gave up the treatment at a subsequent session.

The data analysis consisted initially of descriptive analysis using position and variation measurements (means, medians and standard deviations) and proportions, for the smoking variables, considering the categories of abandonment and non-abandonment of treatment. Subsequently, inferential analysis on the data was carried out using the technique of comparison of two proportions, considering the normal distribution with its respective 95% confidence interval. Therefore, in order to test the difference between these two proportions, the test was used with a significance level of 0.05 (α < 0.05).^19^

To determine whether the data on the six quantitative variables relating to smoking presented normal distribution, the Shapiro test was used. Through this test, it was found that the data did not have normal distribution. To analyze the difference between the group that abandoned treatment and the group that did not abandon it, the nonparametric Mann-Whitney test was used for both categories, comparing the differences between the average levels of the variables relating to the patient’s smoking. In this comparison, the significance level was taken to be 0.05.

Bivariate analysis was conducted, taking the crude prevalence ratio as a reference, with a confidence interval of 95% and significance level of 5% (P < 0.05). The variables with significance levels lower than 20% (P < 0.20), as shown by the chi-square test, were retained for testing in a multiple Poisson regression model with strong variance (RPa), in which the variables that presented P values lower than 5% (P < 0.05) remained in the final model.

The Poisson model was chosen because this has been preferred in the epidemiological literature for estimating the relative risk in cross-sectional or longitudinal studies, using the prevalence ratio.^20^

The dependent variable (outcome) was treatment dropout, and the independent variables considered in the model were gender, age group, motivational level, occupational level, number of years of schooling, CAGE score, psychiatric disorders, physical activity and religion.

This study was submitted to our institution’s Research Ethics Committee on May 9, 2012, under submission certificate (CAAE) no. 0106612.6.0000.5541, and was approved through the committee’s resolution no. 19548.

### RESULTS

**Figure 1.**
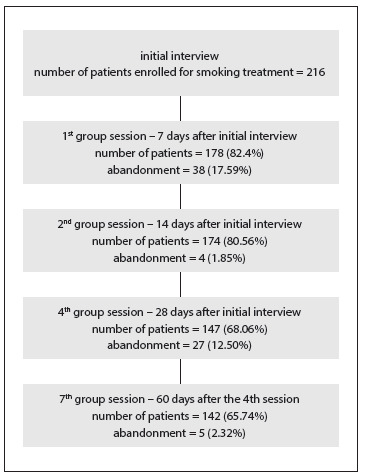
Monitoring of abandonment of treatment among patients during the process.

Out of the 216 initial patients, 74 (34.26%) gave up the treatment during the process, and 142 completed the treatment within six months (65.74%) (Figure 1).

The results from comparisons of the sociodemographic variables, CAGE scores, psychiatric treatments and motivational levels of the participants in both groups (abandonment and non-abandonment) are presented in Table 1. This table shows that there were statistically significant differences (P < 0.05) in the proportions of four variables, for which the P values are highlighted in bold type. These variables are described below.

**Table 1.**
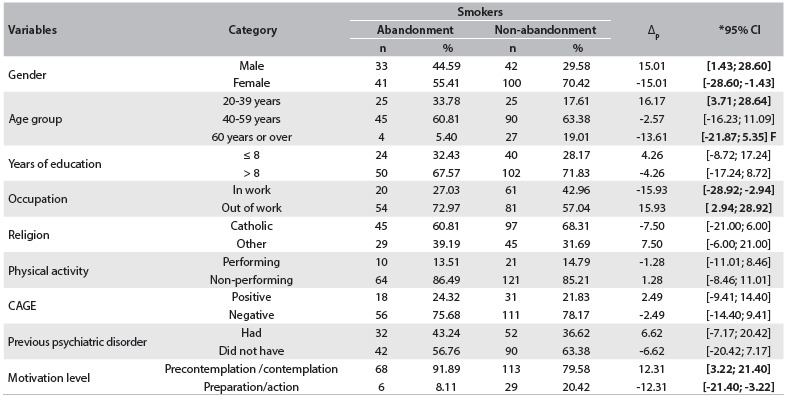
Numbers and percentages of participants, comparing abandonment with non-abandonment of nicotine dependence treatment, according to sociodemographic variables. Cuiabá, Mato Grosso, 2013

Δ_p_ = estimate of the difference in the proportions. Δ_m_ = estimate of the difference in the means. SD = standard deviation. *95% CI = 95% confidence interval for difference in proportions. F = Fisher’s exact test.

Differences were found in relation to gender, age group, occupation and motivational level. For these variables, the categories that most significantly contributed to abandonment and non-abandonment were male gender (P = 0.030 and Δ = 15.01), belonging to the 20-39 year age group (P = 0.011 and Δ = 16.17), having employment (P = 0.016 and Δ = 15.93) and having lower motivational level (P = 0.008 and Δ = 12.31). On the other hand, the variables of number of years of schooling, religion, physical activity, CAGE score and previous psychiatric disorders did not present statistically significant differences between the two groups.

Table 2 presents the average levels of the values for the variables relating to smoking, for the groups that abandoned treatment and did not abandon it. The difference in the average levels of the groups regarding time spent smoking was statistically significant. However, no differences were observed among the groups for the other variables that were studied: smoking history, age when smoking started, number of cigarettes per day, attempts to stop smoking and Fagerström score, taking the significance level to be 0.05.

**Table 2.**
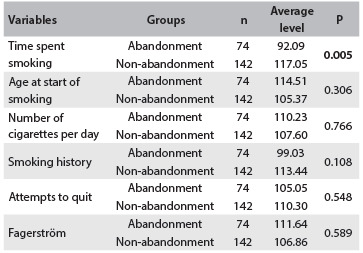
Average levels (*Ri*) of the variable values relating to smoking per group. Cuiabá, Mato Grosso, 2013

P = value associated with the Mann-Whitney test.

The associations between abandonment of treatment of smoking and sociodemographic variables are shown in Table 3. Abandonment was associated with male gender (PR = 1.51; 95% CI: 1.05-2.18), lower age group (PR = 3.88; 95% CI: 1.49-10.08), having employment (PR = 1.62; 95% CI: 1.05-2.50) and motivational levels in the precontemplation and contemplation phases (PR = 2.19; 95% CI: 1.03-4.65). There were no associations between abandonment of treatment and schooling level (P = 0.515), religion (P = 0.270), physical activity (P = 0.80), CAGE score (P = 0.678) or psychiatric disorders (P = 0.343).

**Table 3.**
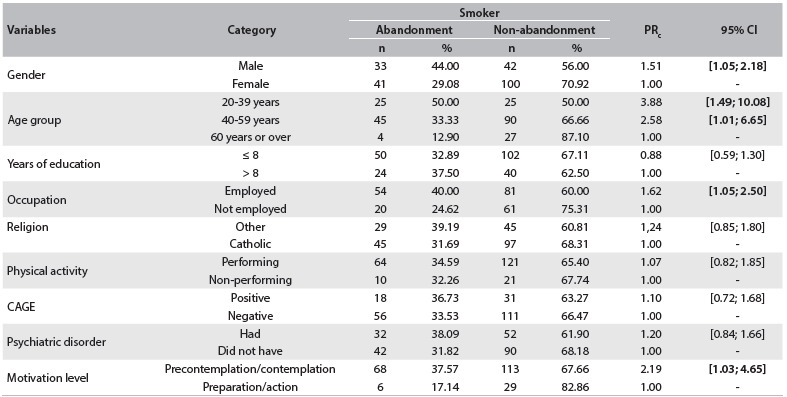
Association between abandonment of smoking and sociodemographic factors. Cuiabá, Mato Grosso, 2013

PR = crude prevalence ratio. 95% CI = 95% confidence interval. P = significance level, considering the chi-square distribution.

Table 4 presents the variables that remained associated with abandonment of treatment after analysis of the final model of robust multivariate Poisson regression. The participants presented greater risk of abandonment of treatment when they were male (RPa = 1.47; 95% CI: 1.03-2.10) and were in the 20 to 39 and 40 to 59 year age groups (RPa = 3.77; 95% CI: 1.47-9.67; and RPa = 2.68; 95% CI: 1.06-6.77).

**Table 4.**
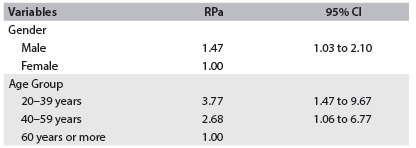
Adjusted prevalence ratio for robust Poisson regression (RPa), for the variables associated with abandonment of smoking among 216 patients, with their respective 95% confidence intervals and the P values for variables selected using the backward method. Cuiabá, MT, 2013

RPa = adjusted prevalence ratio in the Poisson regression model with variable selection; CI = confidence interval.

### DISCUSSION

The rate of abandonment of nicotine dependence treatment in the population studied after six months of observation was high (34.26%), and the majority of the abandonment occurred before CBT was started. These results are similar to those found in other studies.^21^^,^^22^ It is noteworthy that among the participants who were considered to have abandoned their treatment, two groups can be identified: those who did not attend the first CBT session and those who quit during the treatment process. At first glance, these two groups seem to be different from each other; nevertheless, the statistical analysis showed that there were no differences between them regarding the main variables studied here (sociodemographic factors, smoking status, motivational level and CAGE score). Therefore, they were homogeneous and were considered to be a single group, i.e. abandonment.

Some of the variables studied could explain the outcomes that were found, and among these were the sociodemographic characteristics (gender, age group and occupation), those relating to smoking status (duration of tobacco use) and motivational level. It was noteworthy that male participants abandoned treatment more often, thus remaining associated with failure in the final model. There are various plausible explanations for this result. It is a fact that most of the smokers who seek nicotine dependence treatment programs are female.^23^^,^^24^ It is possible that this predominance of females seeking help and then remaining in the programs is due, in large part, to women’s greater concern for their own health, given that there is a difference in health concern depending on gender: women live longer and use healthcare services more frequently than do men, who have a hard time accepting that they are sick.^24^ There are some other important factors that hinder smoking cessation, which the female gender presents with greater intensity: they have more mood disorders, more marked withdrawal syndromes and slower nicotine metabolism; and their weight gain through cessation is more distressing.^24^^,^^25^^,^^26^ Added to this, there is the fact that abandonment was also more prevalent among the participants who were employed. Since males formed the largest workforce and income groups in this population, it is possible that professional commitments were hindering their attendance at the scheduled appointments.^26^^,^^27^ It has been shown that male smokers are embarrassed about being absent from their employment to attend such programs, even if that can obtain a declaration of presence at the healthcare unit.^21^

Another important variable that was associated with the final model for abandonment of treatment was the age group. The youngest group (20-39 years) presented the highest abandonment rate. It is possible to imply that abandonment among young people may have occurred because they still did not feel “sick”, and thought that the harmful effects of smoking would take a long time to appear, such that they would still have plenty of time to decide when to quit definitively.^28^ Studies on licit and illicit drug users have reported that the younger the users are, the lower the chances are that they will remain in treatment.^29^

The degree of success of and adherence to treatment among young individuals presenting substance abuse depends on variables such as severity of dependence and motivation to change behavior, among other things. These two variables proved to be important in the present study. Young people believe that their problems are not related to drugs, and that everything will be all right and nothing bad will happen to them.^30^ Thus, it is necessary to review how to approach these patients in order to achieve higher treatment adherence. This will perhaps not just involve addressing tobacco-related diseases, given that these are still not present and that these individuals have not even envisioned this reality yet. One option would be to work on the esthetic harm caused by chronic use of nicotine (teeth, skin and odor), in an attempt to raise awareness and motivate these young people, who are concerned about their image and appearance.^31^

In relation to the participants in the other age groups, it was observed that they did not abandon treatment so frequently. Despite ambivalence (they felt unable to quit smoking, even though they knew that it was necessary), the older individuals sought support and acceptance, thus suggesting that the decision to seek help occurs at a time of greater maturity, when there is an awareness of the health risks that smoking entails.^26^^,^^32^

Comparisons between the averages relating to occupation and abandonment of dependence treatment should be viewed with caution. Firstly, the results showed that abandonment occurred more often among those who had an occupation, and this was similar to the findings from other studies.^24^^,^^26^ This issue may be related to the times (morning/afternoon) at which controlled visits and program sessions occurred, which are times at which many patients have professional commitments. This may have prevented attendance and may thus have significantly influenced abandonment. The question that arises, therefore, is whether greater schedule flexibility, with appointments in the evenings and/or on weekends, would influence this variable. On the other hand, it is important to remember that the highest smoking prevalence rates are among individuals with lower educational levels who undertake heavy manual labor activities, and these are the very ones who struggle to adhere to extended treatment.^33^

Regarding the variables relating to smoking status, less time spent smoking influenced the abandonment of treatment. This finding shows that the longer the period spent smoking, the more a person will decide to invest in the treatment, perhaps because of the possibility of starting to feel the effects of the diseases caused by tobacco. One of the main reasons why individuals decide to quit smoking relates to the deterioration of health conditions caused by tobacco-related diseases.^34^

Comparison of the average tobacco intake levels showed that the participants who abandoned treatment had lower average levels than those who did not abandon, but the difference was not statistically significant. These results agree with the statement that the higher the dependence is, the greater the search for professional monitoring and the use of medications will be.^35^ Perhaps this reinforces the result in terms of age, in that the younger the age group, the shorter the time spent smoking and the lower the tobacco intake are, the greater the abandonment rate will be. On the other hand, the results found here differ from another study in which dependence levels were not associated with treatment abandonment.^23^

Regarding motivational level, the results show that the participants who were in the precontemplation or contemplation phase were the ones who most abandoned treatment. This result reinforces the established knowledge that individual motivation is the most decisive factor in the process of quitting smoking.^36^ It is known that patients who go to healthcare units for treatment without motivation are a challenge for the therapists, because addictive disorders are essentially motivational.^37^ These results are consistent with those from a study on adolescents who were undergoing treatment for illicit drug use, in which most of those who did not adhere to the program (69.3%) were in the precontemplation phase.^38^

Motivation for change is multifactorial and occurs differently for each human being at given moments in his/her life history.^39^ Perhaps those who do not abandon treatment and stay until the end, even though they do not quit smoking, are patients with high motivational levels. Motivated smokers who are in the preparation or action phase are open to making changes to their behavior, and to taking the necessary steps to do so, and they accept discussion and selection of strategies for the process to be successful.^40^ This motivational level towards quitting smoking encourages smokers to remain in the treatment group, whereas those with low motivational levels (precontemplation or contemplation) tend to abandon the treatment.^41^

Since having a high motivational level is fundamental for non-abandonment and for increasing the chances of adherence to treatment, the whole healthcare team should work on their patients’ motivation. One strategy that has already been tested is the adoption of motivational interviewing, which consists of individualized interventional techniques focused on the patient and tailored to each stage, with the aim of reinforcing the motivation towards change and increasing the treatment adherence.^42^ However, working with motivational interviews requires specialized training for healthcare professionals, because their success depends on the style of those who apply this method, which can directly interfere in the treatment.^43^ Another strategy could be a closer approach to the smoker, so as to stimulate him continuously and show that it is possible to live without smoking and in a healthier way. If necessary, those who did not attend the CBT sessions or the treatment monitoring control appointments could be contacted in person or by telephone.^44^

The major limitation of this study might be the non-characterization of the two groups that initially seemed to be distinct, i.e. the outright abandoners (those who did not even participate in the first session of CBT) and the more resistant individuals (those who abandoned the treatment while it was in progress). It is possible that the number of participants who abandoned the treatment did not allow us to find the differences between them. To further our knowledge of abandonment of nicotine dependence treatment, more studies will be necessary, bearing in mind the distinction between smokers who abandon treatment outright and those who are more resistant.

### CONCLUSIONS

We conclude that in comparing individuals who abandoned nicotine dependence treatment and those who did not abandon it, there are higher dropout rates among male patients and among individuals who belong to the 20-39 age group, have employment, have low motivational levels and have spent shorter periods of time smoking.

Abandonment of treatment for smoking is associated with male gender and a younger age group. Identifying which is the best approach towards dealing with these patients and working with their concerns in order to assist them in carrying out the program is the fundamental key to adherence to treatment of nicotine dependence.
